# The quality of the evidence base for clinical pathway effectiveness: Room for improvement in the design of evaluation trials

**DOI:** 10.1186/1471-2288-12-80

**Published:** 2012-06-18

**Authors:** Thomas Rotter, Leigh Kinsman, Erica James, Andreas Machotta, Ewout W Steyerberg

**Affiliations:** 1 Office of the Dean, School for Public Health and Primary Care (CAPHRI), Faculty of Health, Medicine & Life Sciences, Maastricht University, Maastricht, The Netherlands; 2 School of Rural Health, Monash University, Bendigo, Australia; 3 School of Medicine and Public Health, Priority Research Centre in Health Behaviour, Priority Research Centre for Physical Activity and Nutrition, Hunter Medical Research Institute, University of Newcastle, Newcastle, Australia; 4 Department of Anesthesiology, Sophia Childrens Hospital, Erasmus University Rotterdam, Rotterdam, The Netherlands; 5 Center for Medical Decision Making, Department of Public Health, Erasmus MC – University Medical Center Rotterdam, Rotterdam, The Netherlands

## Abstract

**Background:**

The purpose of this article is to report on the quality of the existing evidence base regarding the effectiveness of clinical pathway (CPW) research in the hospital setting. The analysis is based on a recently published Cochrane review of the effectiveness of CPWs.

**Methods:**

An integral component of the review process was a rigorous appraisal of the methodological quality of published CPW evaluations. This allowed the identification of strengths and limitations of the evidence base for CPW effectiveness. We followed the validated Cochrane Effective Practice and Organisation of Care Group (EPOC) criteria for randomized and non-randomized clinical pathway evaluations. In addition, we tested the hypotheses that simple pre-post studies tend to overestimate CPW effects reported.

**Results:**

Out of the 260 primary studies meeting CPW content criteria, only 27 studies met the EPOC study design criteria, with the majority of CPW studies (more than 70 %) excluded from the review on the basis that they were simple pre-post evaluations, mostly comparing two or more annual patient cohorts. Methodologically poor study designs are often used to evaluate CPWs and this compromises the quality of the existing evidence base.

**Conclusions:**

Cochrane EPOC methodological criteria, including the selection of rigorous study designs along with detailed descriptions of CPW development and implementation processes, are recommended for quantitative evaluations to improve the evidence base for the use of CPWs in hospitals.

## Background

### What are clinical pathways?

Clinical pathways (CPWs) are evidence-based multidisciplinary care plans which describe the essential steps needed in the care of patients with a specific clinical problem. They are used to translate clinical guidelines into local protocols and clinical practice [1]. Whereas clinical guidelines provide generic recommendations, CPWs are specifically tailored to the local hospital structures, systems and time-frames used.

Clinical pathways have been proposed as a strategy to optimise resource allocation in a climate of increasing healthcare costs [2]. Other terms used to describe clinical pathways include ‘integrated care pathways, ’ ‘critical pathways, ’ ‘care plans, ’ ‘care paths’ and ‘care maps.’

#### Objectives

The first objective of this article is to report on the methodological quality of the existing evidence base regarding the effectiveness of CPW research in the hospital setting. An international, multidisciplinary team of researchers conducted a systematic review of the effectiveness of CPWs in hospitals, with the findings recently published in the Cochrane library [3]. The second objective is to test the hypothesis that simple pre-post studies tend to overestimate CPW effects reported.

## Method

We followed the validated Cochrane Effective Practice and Organisation of Care Group (EPOC) methodology for considering and analysing studies [4]. The primary systematic review aimed to catalogue the international evidence to assess the effect of clinical pathways on professional practice, patient outcomes, length of hospital stay and hospital costs. We searched the Database of Abstracts of Reviews of Effectiveness, the Effective Practice and Organisation of Care Register, the Cochrane Central Register of Controlled Trials and bibliographic databases including MEDLINE, EMBASE, CINAHL, NHS EED and Global Health. Details of the electronic search strategy for the identification of studies are presented in detail in the EPOC review, recently published in the Cochrane Library [3]. Our team developed and validated five minimum criteria to define a CPW to ensure that only appropriate studies were sourced and included in the review [5]. An integral component of the review process was a rigorous appraisal of the study designs and methodological quality of all relevant CPW evaluations. This allowed the identification of strengths and limitations of the evidence base for CPW effectiveness with regard to the first study objective.

### Assessment of study design

For the purpose of the systematic review on CPWs in hospitals, four study designs were considered for inclusion: randomized controlled trials (RCTs), controlled clinical trials (CCTs), controlled before and after studies (CBAs) and interrupted time series analysis (ITS).

**Figure 1:**
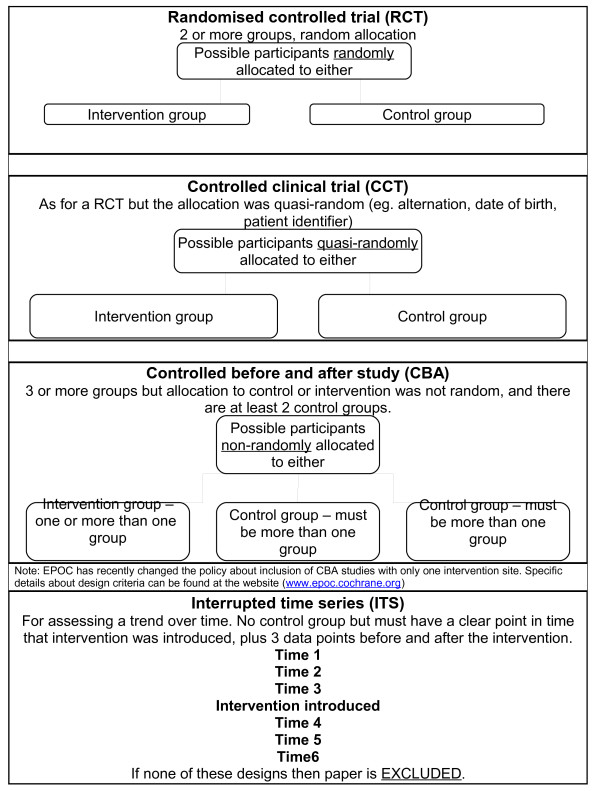
**Simplified EPOC standard of study designs considered for inclusion in the present review.** Source: Bero L, Eccles M, Grimshaw J, Gruen RL, Mayhew A, Oxman AD, Tavender E, Zwarenstein M, Shepperd S, Paulsen E, Pantoja T, Lewin S, Ballini L. Cochrane Effective Practice and Organisation of Care Group (Cochrane Group Module). About The Cochrane Collaboration (Cochrane Review Groups (CRGs)). The Cochrane Library. Oxford: John Wiley, 2009; adopted by the authors.

While there are many well developed and well accepted critical appraisal criteria for experimental studies, fewer exist for non-experimental studies such as CBAs and ITS. Both designs are subject to a lack of control and high risk of bias so EPOC developed criteria to facilitate their quality assessment and inclusion (where appropriate) in systematic reviews. For example, CBAs are required to have more than one control group and ITS require at least three time points before and after an intervention. Validated criteria for the assessment of these designs have been developed by EPOC and are available from the EPOC website [6] and the four different study designs are briefly outlined in Table 1. In addition, the simplified EPOC gold standard of study designs considered for inclusion in the present review are depicted in Figure 1[4].

**Table 1:** EPOC study designs considered for inclusion

Patient randomized controlled trials (P-RCT):	The individual patients are allocated by random to the intervention or control group. Individual randomisation facilitates equally distributed patient characteristics and comparability. Only the exposure to the intervention should be the factor that distinguishes between both groups.
Cluster randomized controlled trials (C-RCT):	This is a robust study design that prevents contamination of professionals by randomising groups of professionals (i.e. different practices, wards or hospitals). However, this means the fundamental assumption of independence is violated because patients within a cluster are more likely to respond in a similar manner. This lack of independence, statistically called “intracluster correlation,” also means a specific adjustment for clustering effects is required to assure comparability with individually randomized trials.
Non-randomized controlled trials (CCTs):	Patient or cluster trials where allocation to experimental and control groups is quasi-random (i.e. alternated allocation).
Controlled before and after studies (CBAs):	CBAs are experimental studies with two or more control groups compared with one or more experimental groups but allocation is not random. Data is collected on the control and intervention groups before the intervention is introduced and then further data is collected after the intervention has been introduced. The reliability of the intervention effect is questionable because there may be unidentified differences between the experimental intervention and control groups which may have modified the observed effect. Note: EPOC has recently changed the policy about inclusion of CBA studies with only one intervention site. Specific details about design criteria can be found at the website (http://www.epoc.cochrane.org)
Interrupted time series designs (ITS):	This represents a robust method of measuring the effect of an intervention as a trend over time. It is a useful design when recruitment of a control cohort is impractical, e.g. due to changes in hospital policy. Three or more data points are collected before and after the intervention as a minimum standard. The intervention effect is measured against the pre-intervention trend.

Source: Bero L, Eccles M, Grimshaw J, Gruen RL, Mayhew A, Oxman AD, Tavender E, Zwarenstein M, Shepperd S, Paulsen E, Pantoja T, Lewin S, Ballini L. Cochrane Effective Practice and Organisation of Care Group (Cochrane Group Module). *About The Cochrane* Collaboration (Cochrane Review Groups (CRGs)). The Cochrane Library. Oxford: John Wiley, 2009; adopted by the authors.

### Risk of bias assessment

We developed a quality assessment and data abstraction instrument incorporating the EPOC risk of bias criteria [4]. Quality assessment was conducted on full-text articles once initial literature searching and screening indicated that articles were research-based and referred to a CPW and were, subsequently, potentially relevant. The EPOC approach for judging risk of bias of randomized and non-randomized studies is a two-part assessment tool, concerning specific domains and quality criteria (i.e. RCTs: sequence generation, allocation concealment, blinding, et cetera). The validated risk of bias criteria can be found in the Cochrane EPOC Group module [4] and are presented in detail in additional file 1.

### Comparison of CPW interventions

We compared patients managed according to CPW to those managed by usual care, and patients treated within a multifaceted intervention including a CPW compared to usual care.

### Secondary analysis

The aim of the secondary analysis was to determine whether pre-post study design was associated with an overestimate of the effects of CPW. Other researchers also compared the findings of randomized evaluations vs. non-randomized study designs and concluded that such studies potentially overestimate the effects reported and there were systematic differences between effects estimated [7-9]. To test the hypothesis, we compared 14 primary studies [10-23], included in the Cochrane review, grouped into category 1 (patients managed according to CPW compared to usual care), and reporting on length of stay (LOS) as the most commonly employed outcome measure with a randomly selected sample of 14 excluded pre-post CPW evaluations also reporting LOS [24-37]. The selection of a random sample of studies was taken from those studies excluded on the basis of a simple pre-post design not meeting EPOC quality criteria (see Table 2). We used a computer generated random sample (RAND function in Excel) [38] of 14 excluded pre-post studies reporting LOS as a primary study outcome [24-37].

**Table 2:** Reasons for exclusion stage one (n = 2954)

**Reason**	**Number**	**%**
Not CPW	2335	79.1
Not study	253	8.6
Not hospital	246	8.3
EPOC minimum study design criteria not met	89	3.0
Other (e.g. qualitative study)	31	1.0
Total	2954	100

#### Statistical pooling (meta-analysis)

A Cochrane web-based program, Review Manager (RevMan), was used to calculate a pooled estimate of the combined intervention effect on LOS, called weighted mean difference (WMD) [39]. We used a random effects model since this model estimates the effect with consideration to the variance between studies, rather than ignoring heterogeneity by employing a fixed effect model [40]. Statistical inconsistency within both subgroups was assessed by calculating a test of heterogeneity (I square (I^2^)).

## Results

**Figure 2:**
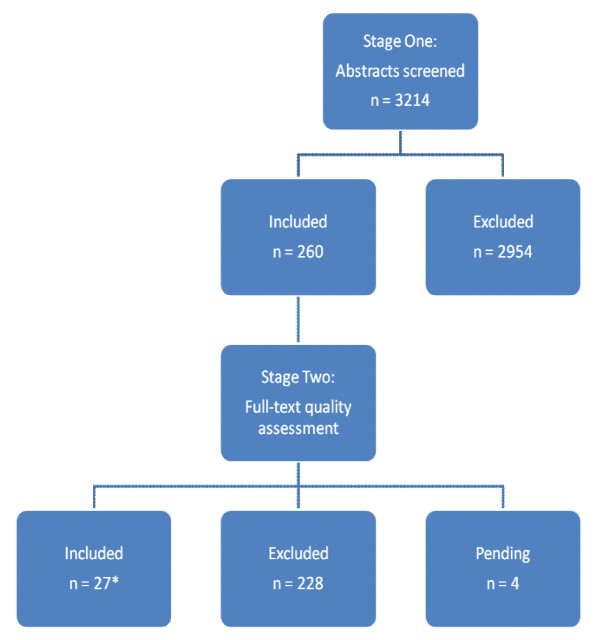
Trial flow.

All potentially relevant studies were assessed using the CPW definition [5] and EPOC review inclusion criteria for acceptable study designs [4]. Using two independent reviewers, we rejected 2954 of the 3214 potential papers and only 260 primary studies were initially identified as potentially relevant and full text copies were retrieved. Figure 2 illustrates the described trial flow.

The majority (79.1 %) of the rejected studies (2335 out of 2954) had to be excluded because they failed to meet our definition of CPW. Table 2 illustrates the reasons for exclusion following title and abstract review. Out of the 260 primary studies meeting CPW content criteria following review of the full text, only 27 studies met the EPOC study design and risk of bias criteria.

### Assessment of study design

Out of 27 CPW evaluations included, nineteen of the included studies were randomised controlled trials (RCTs) [10,11,13-22,41-48], including two cluster randomised trials (C-RCT) [20,47]. Four studies were CBAs [49-52], two were CCTs [12,23] and two ITS [53,54].

Of the original studies which met the CPW content criteria, more than 70 % were excluded from the review as they were simple pre-post evaluations, mostly comparing two or more yearly patient cohorts (see Table 3).

**Table 3:** Reasons for exclusion following full text review (n = 228)

**Reason**	**Number**	**%**
Not CPW	38	16.7
Simple pre-/post evaluations	160	70.2
High risk of bias	5 (1RCT)	2.2
Not study	14	6.1
Not hospital	11	4.8
Total	228	100

### Risk of bias assessment

Out of the 228 studies excluded in phase two following full text review (see Table 3) only four non-randomized studies [55-58] and one randomised clinical study (RCT) [59] were excluded because of high risk of bias. The RCT from Bittinger (1995) did not meet EPOC quality criteria as only 50 % of study patients were followed up after randomization and there was a high risk of attrition bias. Four time series studies were excluded as data was not analyzed appropriately. The studies from Joiner (1996), Smith (1999), Summers (1998) and Warner (2002) had a high risk of bias because no statistical control was used [55-58].

Table 3 illustrates the reasons for exclusion in stage two after meeting CPW content criteria in stage one.

### Secondary analysis

**Figure 3:**
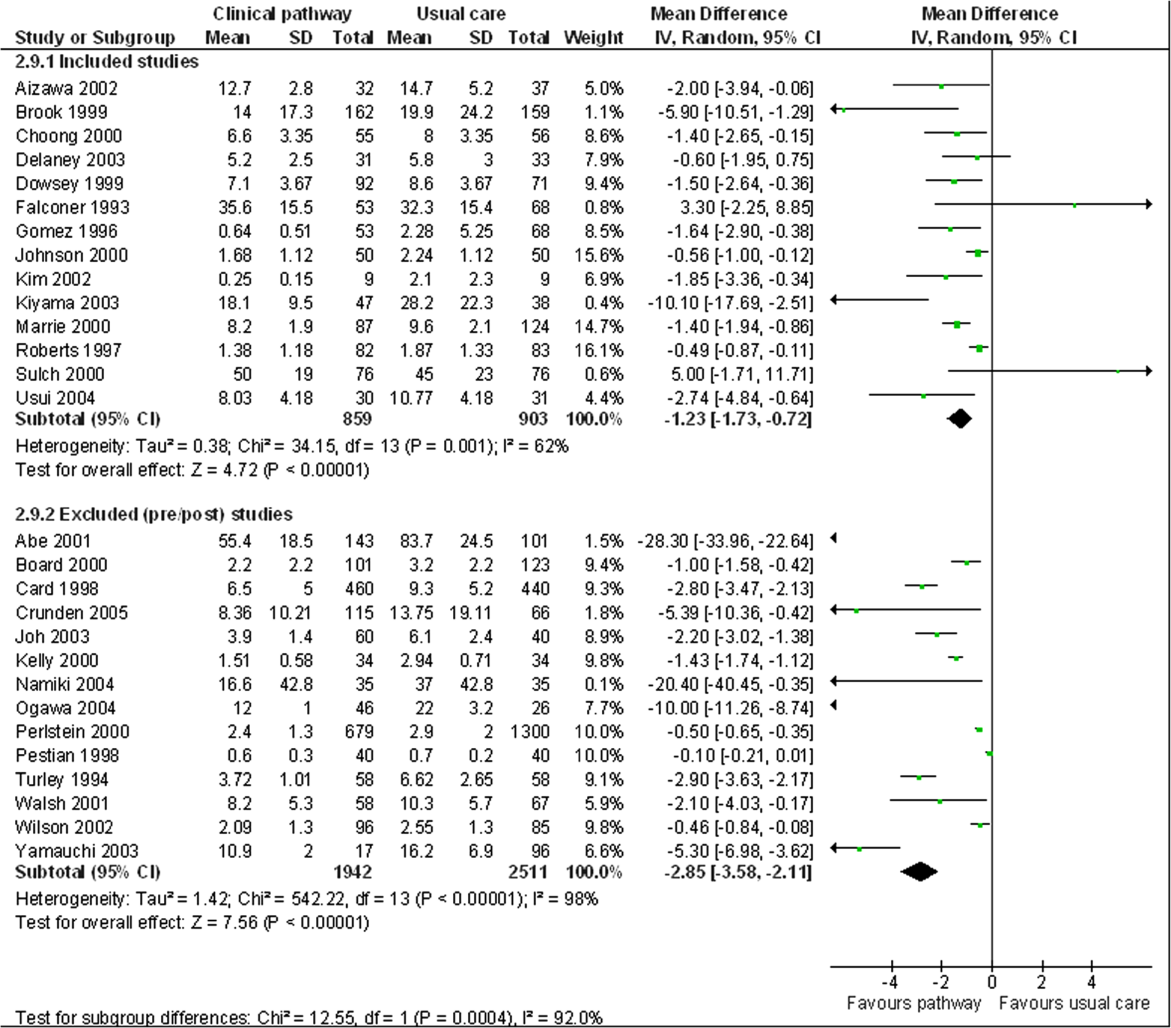
Comparison meta analysis LOS COCHRANE vs. Excluded pre-post studies.

In Figure 3 we provide the detailed results of the methodological comparison of the 14 included primary studies which utilised Cochrane EPOC study design quality criteria [4] and reporting on LOS [10-23] vs. 14 randomly selected pre-post studies excluded from the review and reporting on LOS as a primary outcome [24-37]. We observed considerable statistical inconsistency within both subgroups of CPW studies, so the calculated estimates in LOS per subgroup should be treated with caution (I² = 62% Cochrane EPOC subgroup vs. 98% randomly selected subgroup.)

We observed greater reported LOS effects within the random subgroup of excluded pre-post studies after meeting CPW content criteria (WMD – 2.85 (95%CI: – 3.58 to – 2.11)), versus the pooled LOS data recently published in the Cochrane library (group 1 clinical pathway vs. usual care WMD – 1.23 (95%CI – 1.73 to – 0.72)) [3]. Moreover, the pre-post studies in the randomly selected subgroup tend to report more consistently on significant reductions in LOS (see Figure 3). Statistically, the chi-squared test for subgroup differences also reached a significant level (P = 0.0004).

## Discussion

### Why is it important to critically appraise study designs in a systematic review?

We followed the validated Cochrane EPOC criteria for randomized and non-randomized CPW evaluations [4,6]. The finding that the vast majority of studies failed to meet methodological quality criteria strongly indicates that low quality study designs are too often used to evaluate CPWs and contribute very little to the evidence base regarding CPWs.

Many of such excluded CPW evaluations claimed to provide evidence for the effectiveness of the pathway intervention under consideration but, with a methodologically weak study design, it remains unclear if the reported effect was really attributable to the CPW effectiveness or any other unknown factors. Possible confounding factors might have been the case-mix introduction, hospital quality improvement initiatives or changes in hospital policy [2]. The uncontrolled nature and exposure to bias convey that such studies contribute very little to the evidence-base.

### Implications of including weak study designs

Based on our review experience, we reaffirm that uncontrolled pre-post designs are commonly used to evaluate the effectiveness of CPWs. Such designs are likely to be misleading and contribute little to understanding the reported effects of pathways. Considering the second objective of this article, the meta-analytic comparison supports other evidence [7-9] that simple pre-post study designs tend to overestimate intervention effects reported.

There is a place for well designed process-evaluations also referred to as interrupted time series (ITS) to explore and provide more insights into the varying pathway components and their causal effectiveness to determine how CPW interventions actually work. Carefully designed time series studies are less resource-intensive than RCTs, do not require a control group, and allow for the use of retrospective data. While requiring more advanced statistical techniques than simple pre-post studies, ITS supports research outcomes that are more likely to contribute to the evidence base, including systematic reviews. Better designed, conducted and reported CPW evaluations will contribute to a better understanding of the key elements of CPWs that impact on patient, provider and economic outcomes.

### Limitations

The majority of included studies employed LOS as a performance measure. Hence, we compared the magnitudes of CPW effects on length of stay (n = 14 primary studies) rather than patient outcomes such as mortality (n = 4 studies) or in-hospital complications (n = 5 studies) [3]. The low number of primary CPW evaluations included in the review which reported on patient outcomes prevented further testing of the robustness of this methodological comparison.

## Conclusion

Cochrane EPOC methodological inclusion criteria should be considered for quantitative evaluations into the impact of CPWs in hospitals. Based on our review experience, the EPOC methodological gold standard is infrequently transferred into research practice. Future evaluators could hereby contribute significantly to the understanding of factors associated with the reported effects of clinical pathways in hospitals by incorporating EPOC criteria into study design. Whilst experimental methods such as randomised trials are recommended they may be considered beyond the capacity of many clinicians and researchers. A well designed evaluation such as ITS or CBA that meets the EPOC gold standard methodological criteria can produce meaningful, rigorous results with the use of relatively few resources. In terms of the second study objective, the methodological comparison of Cochrane vs. non Cochrane study designs (see Figure 3) also support the finding that simple pre-post study designs tend to overestimate CPW effects reported.

## Competing interests

The authors declare that they have no competing interests.

## Authors’ contributions

TR, LK, EJ and AM participated equally to the development and preparation of the manuscript. TR, LK and EWS contributed to study design and writing of the manuscript. All authors read and approved the final manuscript.

## Pre-publication history

The pre-publication history for this paper can be accessed here:

http://www.biomedcentral.com/1471-2288/12/80/prepub

## Supplementary Material

Additional file 1 EPOC risk of bias criteria. Click here for file
