# Supplementary material for: The quality of the evidence base for clinical pathway effectiveness: Room for improvement in the design of evaluation trials
Source: BMC Med Res Methodol. 2012 Jun 18;12:80. doi: 10.1186/1471-2288-12-80 (PMC3424110; doi:10.1186/1471-2288-12-80)
Supplement: Additional file 1 — EPOC risk of bias criteria. [file 1471-2288-12-80-S1.doc]

Additional table 1: risk of bias criteria for EPOC reviews

| Risk of bias for studies with a separate control group  Randomised controlled trials (RCTs)  Controlled clinical trials (CCTs)  Controlled before-after (CBA) studies  Nine standard criteria are used for all RCTs, CCTs and CBAs. Further information can be obtained from the Cochrane handbook section on Risk of Bias and from the draft methods paper on risk of bias under the EPOC specific resources section of the EPOC website. |
| --- |
| Was the allocation sequence adequately generated?  Score “Yes” if a random component in the sequence generation process is described (eg Referring to a random number table). Score ”No” when a nonrandom method is used (eg performed by date of admission). NRCTs and CBA studies should be scored “No”. Score “unclear” if not specified in the paper. |
| Was the allocation adequately concealed?  Score “Yes” if the unit of allocation was by institution, team or professional and allocation was performed on all units at the start of the study; or if the unit of allocation was by patient or episode of care and there was some form of centralised randomisation scheme, an on-site computer system or sealed opaque envelopes were used. CBA studies should be scored “No”. Score “unclear” if not specified in the paper. |
| Were baseline outcome measurements similar?  Score “Yes” if performance or patient outcomes were measured prior to the intervention, and no important differences were present across study groups. In RCTs, score “Yes” if imbalanced but appropriate adjusted analysis was performed (e.g. Analysis of covariance). Score “No” if important differences were present and not adjusted for in analysis. If RCTs have no baseline measure of outcome, score “Unclear”. If some primary outcomes were imbalanced at baseline, assessed blindly or affected by missing data and others were not, each primary outcome can be scored separately. If “UNCLEAR” or “No”, but there is sufficient data in the paper to do an adjusted analysis (e.g. Baseline adjustment analysis or Intention to treat analysis) the criteria should be re scored as “Yes”. |
| Were baseline characteristics similar?  Score “Yes” if baseline characteristics of the study and control providers are reported and similar. Score “Unclear” if it is not clear in the paper (e.g. characteristics are mentioned in text but no data were presented). Score “No” if there is no report of characteristics in text or tables or if there are differences between control and intervention providers. Note that in some cases imbalance in patient characteristics may be due to recruitment bias whereby the provider was responsible for recruiting patients into the trial. |
| Were incomplete outcome data adequately addressed?  Score “Yes” if missing outcome measures were unlikely to bias the results (e.g. the proportion of missing data was similar in the intervention and control groups or the proportion of missing data was less than the effect size i.e. unlikely to overturn the study result). Score “No” if missing outcome data was likely to bias the results. Score “Unclear” if not specified in the paper (Do not assume 100% follow up unless stated explicitly). If some primary outcomes were imbalanced at baseline, assessed blindly or affected by missing data and others were not, each primary outcome can be scored separately. |

(additional table 1: continued)

| Was knowledge of the allocated interventions adequately prevented during the study?  Score “Yes” if the authors state explicitly that the primary outcome variables were assessed blindly, or the outcomes are objective, e.g. length of hospital stay. Primary outcomes are those variables that correspond to the primary hypothesis or question as defined by the authors. Score “No” if the outcomes were not assessed blindly. Score “unclear” if not specified in the paper. If some primary outcomes were imbalanced at baseline, assessed blindly or affected by missing data and others were not, each primary outcome can be scored separately. |
| --- |
| Was the study adequately protected against contamination?  Score “Yes” if allocation was by community, institution or practice and it is unlikely that the control group received the intervention. Score “No” if it is likely that the control group received the intervention (e.g. if patients rather than professionals were randomised). Score “unclear” if professionals were allocated within a clinic or practice and it is possible that communication between intervention and control professionals could have occurred (e.g. physicians within practices were allocated to intervention or control) |
| Was the study free from selective outcome reporting?  Score “Yes” if there is no evidence that outcomes were selectively reported (e.g. all relevant outcomes in the methods section are reported in the results section). Score “No” if some important outcomes are subsequently omitted from the results. Score “unclear” if not specified in the paper. |
| Was the study free from other risks of bias?  Score “Yes” if there is no evidence of other risk of biases |
| **Risk of bias for interrupted time series (ITS) studies**  Seven standard criteria are used for all ITS studies. Further information can be obtained from the EPOC specific resources section of the EPOC website.    Note: If the ITS study has ignored secular (trend) changes and performed a simple t-test of the pre versus post intervention periods without further justification, the study should not be included in the review unless reanalysis is possible. |
| Was the intervention independent of other changes?  Score “Yes” if there are compelling arguments that the intervention occurred independently of other changes over time and the outcome was not influenced by other confounding variables/historic events during study period. If Events/variables identified, note what they are. Score “NO” if reported that intervention was not independent of other changes in time. |
| Was the shape of the intervention effect pre-specified?  Score ”Yes” if point of analysis is the point of intervention OR a rational explanation for the shape of intervention effect was given by the author(s). Where appropriate, this should include an explanation if the point of analysis is NOT the point of intervention; Score “No” if it is clear that the condition above is not met |

(additional table 1: continued)

| Was the intervention unlikely to affect data collection?  Score “Yes” if reported that intervention itself was unlikely to affect data collection (for example, sources and methods of data collection were the same before and after the intervention); Score “No” if the intervention itself was likely to affect data collection (for example, any change in source or method of data collection reported). |
| --- |
| Was knowledge of the allocated interventions adequately prevented during the study?  Score “Yes” if the authors state explicitly that the primary outcome variables were assessed blindly, or the outcomes are objective, e.g. length of hospital stay. Primary outcomes are those variables that correspond to the primary hypothesis or question as defined by the authors. Score “No” if the outcomes were not assessed blindly. Score “unclear” if not specified in the paper. If some primary outcomes were assessed blindly or affected by missing data and others were not, each primary outcome can be scored separately. |
| Were incomplete outcome data adequately addressed?  Score “Yes” if missing outcome measures were unlikely to bias the results (e.g. the proportion of missing data was similar in the pre- and post-intervention periods or the proportion of missing data was less than the effect size i.e. unlikely to overturn the study result). Score “No” if missing outcome data was likely to bias the results. Score “Unclear” if not specified in the paper (Do not assume 100% follow up unless stated explicitly). If some primary outcomes were assessed blindly or affected by missing data and others were not, each primary outcome can be scored separately. |
| Was the study free from selective outcome reporting?  Score “Yes” if there is no evidence that outcomes were selectively reported (e.g. all relevant outcomes in the methods section are reported in the results section). Score “No” if some important outcomes are subsequently omitted from the results. Score “unclear” if not specified in the paper. |
| Was the study free from other risks of bias?  Score “Yes” if there is no evidence of other risk of biases.  e.g. should consider if seasonality is an issue (i.e. if January to June comprises the pre-intervention period and July to December the post, could the “seasons’ have caused a spurious effect). |
| Source: Bero L, Eccles M, Grimshaw J, Gruen RL, Mayhew A, Oxman AD, Tavender E, Zwarenstein M, Shepperd S, Paulsen E, Pantoja T, Lewin S, Ballini L. Cochrane Effective Practice and Organisation of Care Group (Cochrane Group Module). *About The Cochrane* Collaboration (Cochrane Review Groups (CRGs)). The Cochrane Library. Oxford: John Wiley, 2010; adopted by the authors |
